# Editorial: Sport and the politics of in/equality

**DOI:** 10.3389/fsoc.2022.1087572

**Published:** 2023-01-06

**Authors:** Elizabeth C. J. Pike, Andy Smith

**Affiliations:** ^1^Institute of Sport, University of Hertfordshire, Hatfield, United Kingdom; ^2^Department of Sport and Physical Activity, Edge Hill University, Ormskirk, United Kingdom

**Keywords:** sport, politics, inequality, sociology, policy

There is increasing global interest among social scientists of the widening social inequalities which characterize many societies across the world. Interest in the significance of social inequalities related to divisions such as class, gender, age, race and ethnicity, place and location, sexuality, ability, religion, identity, and the intersections between these, is not new. Sociologists have long been interested in how they are experienced, how they shape life chances, and how they help make sense of social relationships. However, the scale and pace of growth in various forms of inequality in the last decade or so, many of which have been exacerbated by COVID-19, has rekindled interest in the corrosive effects of inequality on individuals, communities, and whole societies not only among academics, but also policy-makers, politicians, world leaders, the media, and those whose lives continue to be affected by them.

The politics of equality and inequality is also of interest among sociologists of sport, since it is well-established that sports are social phenomena that reflect, reveal, reproduce, and reinforce social inequalities, ideologies, and social exclusion. Sports are also potential sites of resistance to social inequality, of producing social change, of improving life chances, and of fostering social relationships which provide the basis for a more humane social world.

The papers in this Research Topic bring together bodies of knowledge in sociology, sport, politics, and policy analysis to examine the production and reproduction of social inequalities, and of the potential challenges to them, in and through the various relationships and processes which constitute sport. In particular, the papers examine inequalities in contemporary social relations which exist in sport focusing on the experiences of women, transgender persons, young people, and older adults. The papers are informed by a range of theoretical approaches including Marxism, Feminism, Bourdieu, and Putnam. The data collected draws from policy reviews, interviews, observation, and co-production methods. The findings demonstrate the significance of ideology, policy, community, and place when understanding how sport can be both a cause of, and solution for, social inequalities, as well as providing sites for promoting health and wellbeing, quality of social relationships, social capital, and trust.

Burnett's paper analyses policy frameworks in the context of post-Apartheid South Africa to consider the ways in which people “do gender” as part of the multiple layers of relative disenfranchisement which also includes class and race. The article demonstrates the continued impact of patriarchal ideology, hegemonic gendered culture, significant others, and the availability of resources and democracy on the lives of women and girls, particularly in terms of barriers to participation in, and leadership of, sport. Burnett makes the case for structural and systemic reform to enable full gender inclusivity and empowerment in sport and society.

Pike et al. evaluate a community-led physical activity programme for young people in the United Kingdom. Their findings demonstrate the potential of such programmes for improving levels of physical activity, while also offering the potential for improving relations within neighborhoods and developing a sense of community. The paper argues for the significance of social capital as both cause and effect for successfully influencing policy, and the need to combine social capital with elements of social movements to ensure sustained outcomes.

In the third article, Mansfield et al. critically evaluate the disconnect between research, policy and the promotion of physical activity for older adults. They draw on findings from a coproduced community sport project for older adults with limited income. Their findings demonstrate the need to move beyond focusing on practical barriers to consider the intersections of biological, psychological, and social experiences of older adults to develop a fuller understanding of the complexity and inequality which impact on engagement in, and enjoyment of, physical activity.

The final paper in this Research Topic is by Caudwell and centers on the transgender and non-binary experiences of recreational swimmers. The article examines the impact of feeling unsafe in sports spaces and, in particular, the display of embodiment in public swimming pools. These impact on the experiences of physical activity and also the production of inequality in and through sport. Caudwell demonstrates the potential for such research to inform evidence-based interventions to address social inequality.

Collectively the papers draw critical attention to the various ways in which social inequalities intersect and shape experiences of sport and physical activity participation among particular social groups, and how these experiences simultaneously express, reinforce and exacerbate existing patterns of inequalities in engagement in sport and physical activity. The papers also reveal how participating in community-based programmes can produce various social and health outcomes, including the promotion of opportunities for people to be physically active, but that their impact is limited by the pervasive effects of the deeply entrenched social inequalities which characterize those communities and the societies of which they are a part. As the scale of social inequalities continue to widen across the world, and within individual and between individual countries, the findings of the papers included in this Research Topic will provide an important starting point for future research which investigates the complex relationships between sport, the politics of inequality and our wellbeing.

## Author contributions

Both authors listed have made a substantial, direct, and intellectual contribution to the work and approved it for publication.

